# Exploring the landscape of palliative care provision for black patients with hematologic cancers: A scoping review

**DOI:** 10.1017/S1478951525000471

**Published:** 2025-04-22

**Authors:** Osborn Owusu Ansah, Silas Selorm Daniels-Donkor, Leila Ledbetter, Matthew LeBlanc, Sophia K. Smith

**Affiliations:** 1 Duke University School of Nursing, Durham NC, USA; 2 University of Virginia, School of Nursing, Charlottesville, VA, USA; 3 Duke University Medical Center Library, Durham, NC, USA; 4 University of North Carolina, School of Nursing, Chapel Hill, NC, USA; 5 Duke Cancer Institute, Durham, NC, USA

**Keywords:** Black patients, hematologic malignancies, palliative care, scoping review, racial inequities

## Abstract

**Objectives:**

Patients with cancer benefit greatly from receiving palliative care (PC), improving their overall survival and quality of life. Despite its benefits, PC is underutilized among patients with hematologic malignancies (HMs), particularly among Black patients, who face higher symptom burdens and lower survival rates compared to White patients. The purpose of this review was to identify and describe what is known about PC use among Black HM patients in the United States.

**Methods:**

This review was conducted using the Joanna Briggs Institute approach for scoping reviews and included a search of the databases MEDLINE (PubMed), Embase (Elsevier), Scopus and Web of Science (Clarivate). The search was developed and conducted by a professional medical librarian in consultation with the author team and focused on keywords such as Black/African American patients, HM, and PC. Articles were screened and selected based on predefined inclusion criteria and carried out using Covidence software for systematic review management.

**Results:**

Seven publications were included in the final sample and most used quantitative methods and data from large national databases such as the National Cancer Database. Four of the studies reported that Black patients with HM were less likely to receive or use PC compared to White patients. Access to PC was associated with better outcomes, such as lower hospital charges and a reduced likelihood of dying within 30 days of initiating palliative radiotherapy.

**Significance of the results:**

This scoping review highlights ongoing inequities in the use of PC among Black patients with HM which mirrors trends in patients with solid cancers. Future studies should be conducted to understand the determinants of these disparities and to also build testable interventions to improve PC use within this underserved population.

## Introduction

Palliative care (PC) is a specialized medical discipline that has become an integral part of care for patients with cancer (Tan and Ramchandran 2020). The evidence indicates that incorporating PC into standard oncologic treatment can improve the quality of life and satisfaction for both patients and their caregivers (Dionne-Odom et al. 2015; McDonald et al. 2016). PC specialists provide symptom management through expert symptom monitoring, sufficient patient-clinician communication and establishment of treatment plans that can address all facets of the patients’ needs (Senderovich and McFadyen 2020). For patients with hematologic malignancies (HMs), receiving PC is particularly beneficial considering the high physical and psychological symptom burden these patients often experience from complications due to the disease and side effects from the intensive treatments (El-Jawahri et al. 2020; Kayastha and LeBlanc 2022). Studies demonstrate that patients with HM commonly present with severe fatigue, poor well-being, bodily pain, weight loss, anxiety, and depression (Boswell et al. 2023; Newcomb et al. 2020). Furthermore, incorporation of early PC can reduce treatment costs and provide an opportunity for more holistic patient care (LeBlanc et al. 2018).

Although the evidence supports the inclusion of PC into standard oncology care, PC remains underused among patients with HM (El-Jawahri et al. 2020; Mohyuddin et al. 2020). According to reports, patients with HM frequently receive aggressive end-of-life care, which includes a higher risk of hospital death, toxic chemotherapy, and poor hospice use (LeBlanc and El-Jawahri 2018; Resick et al. 2020). Despite the evident inequities in the use of PC among most patients with HM, it is important to understand if there are racial differences in the utilization of PC, particularly among Black patients who may face additional barriers to accessingthese services. Black patients with HM experience significantly longer delay to receiving intensive and efficacious treatments such as stem cell transplantation (Bhatnagar et al. 2015). In addition, Black patients with HM have worse survival outcomes when compared to their White counterparts (Kirtane and Lee 2017). This is concerning given that Black patients have a lower overall five-year survival rate for most cancers, including HM (American Cancer Society 2022).

The PC guidelines from the American Society of Clinical Oncology on integrating health equity indicate that there is a critical need for research data on these populations to tailor interventions and ensure cultural sensitivity and inclusion in PC delivery for underserved populations (Rosa et al. 2024). Therefore, the objective of this narrative scoping review is to identify and describe what is known about PC use among Black HM patients in the United States. This review contributes to the efforts aimed at reducing barriers and promoting the uptake of PC services within the Black HM community. The findings of this scoping review could also inform the design of future interventions aimed at promoting and ensuring that PC is administered with cultural understanding and sensitivity.

## Methods

### Design

This scoping review was conducted using the Joanna Briggs Institute approach for scoping reviews (Peters et al. 2020) and reported following the Preferred Reporting Items for Systematic Reviews and Meta-Analysis (PRISMA-ScR) extension for Scoping Reviews checklist (Tricco et al. 2018). We registered the protocol for this review in the Open Science Framework database (https://doi.org/10.17605/OSF.IO/75CAJ).

### Information sources

The databases searched included MEDLINE (PubMed), Embase (Elsevier), CINAHL Complete (EBSCOhost and Web of Science (Clarivate). We selected those four databases because the Cochrane Handbook (section 4.3.1.1) advises that the search for studies for a review should be as thorough as possible to lower the risk of reporting bias and to find as much relevant information as feasible (Lefebvre et al. 2025). In addition, searching two or more databases reduces the risk of missing eligible studies (Ewald et al. 2022).

### Search strategy

A medical librarian (L.L.) worked with the author team to plan and carry out the searches, which were based on three (3) key ideas: Black/African American, PC, and HMs. For each database, publication types like editorials, letters, comments, and conference abstracts were removed using search hedges or database filters. The search was conducted on June 24, 2024, and found 443 citations. An updated search was conducted on October 24, 2024, and no new citations were found to be included for further review. The Supplementary Materials contain comprehensive replicable search techniques for every database, including date ranges and search filters. A reference-list review of the final included articles was carried out by a study team member (OOA) to identify additional relevant evidence, and no further relevant articles were identified.

### Eligibility criteria

We included publications that met the following criteria: (1) the sample included adult Black/African American with HMs (18 years and above); (2) a focus on PC strategies, interventions, or models of care including but not limited to end of life care, hospice care, palliative radiotherapy and palliative communication; and (3) employed qualitative, quantitative and/or mixed methods in a study conducted within the US. We excluded articles that focused on: (1) pediatric populations; (2) not published in English; and (3) opinion pieces, editorials, case reports, and systematic or scoping reviews.

### Study selection

**Figure 1.**
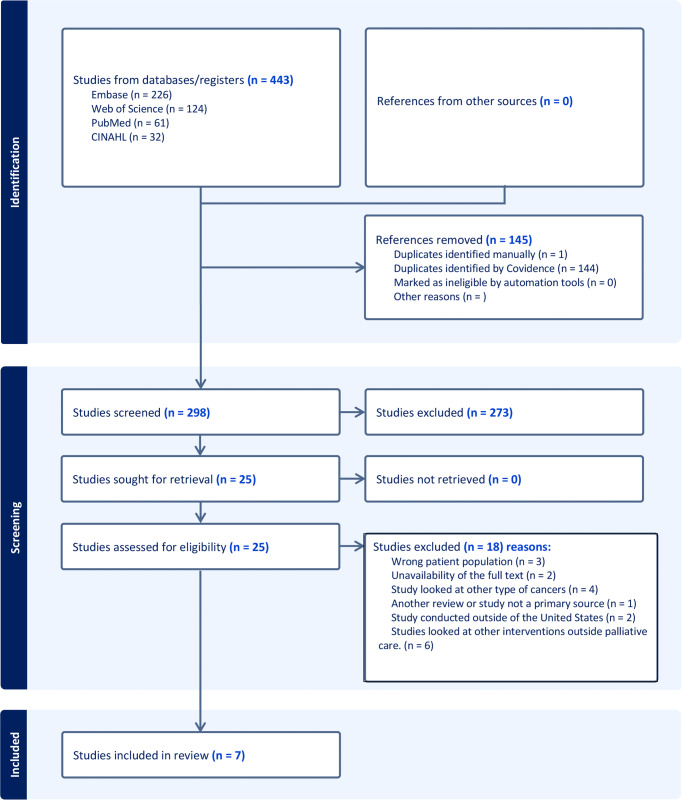
PRISMA diagram for new systematic reviews which includes searches of databases and other sources.

After the search process, the resulting citations were uploaded into Covidence (Veritas Health Innovation, Melbourne, Australia), a software system for managing systematic reviews, and 144 duplicates were removed by the software before screening. The title/abstract phase involved screening the remaining 299 citations. Both the title/abstract screening and the full text screening were performed independently by two reviewers (O.O.A. and S.S.D.). After a thorough analysis, articles that did not meet the eligibility criteria were excluded. Any conflicts between the reviewers were resolved through discussion among the reviewers. The article selection process is presented by flowchart per PRISMA guidelines (Figure 1).

### Data extraction

We developed a customized data extraction chart in EXCEL and later transferred into a word document with the following sections: (1) authors and year, (2) study aim, (3) design, (4) study population, (5) characteristics of Blacks/African Americans participants included in the study, (6) HM type studied and (7) significant findings. All reviewers participated in a pilot testing of the data extraction template, which was then modified based on the input from the team. Data extraction was first done independently by one reviewer (O.O.A.) and verified in full by a second reviewer (S.S.D.). The differences in data between the two authors were discussed and resolved by all authors. The final data was discussed and approved by all four authors.

### Synthesis of the evidence

Following extraction, the primary findings from the various evidence sources were examined. Through a discussion among the study team, the main findings were identified and described as per the aim of the review. The topics served as the basis for the narrative synthesis. All team members were involved in this process, and any disagreements were discussed to resolve any conflicts.

## Results

Seven studies met the inclusion criteria after the full-text screening and were subsequently included in this review.

### Study characteristics

All the included articles employed quantitative methods and were published between 2019 and 2024. Six were cohort studies (Al Hadidi et al. 2021; Chan et al. 2024; Fossum et al. 2021; Han et al. 2019; Hsieh et al. 2024; Rao et al. 2021), and one was a cross-sectional study (Jackson et al. 2022). All the studies used samples generated from population-specific databases such as the National Cancer Database (Chan et al. 2024; Fossum et al. 2021), the National Inpatient Sample (Al Hadidi et al. 2021; Han et al. 2019; Hsieh et al. 2024; Jackson et al. 2022), and the National Cancer Institute’s Surveillance, Epidemiology, and End Results-Medicare linked database (Rao et al. 2021).

Three of the 7 studies included participants who were diagnosed with multiple myeloma (Al Hadidi et al. 2021; Fossum et al. 2021; Jackson et al. 2022), 2 studies included a variety of HM such as leukemia, lymphoma, myelodysplastic syndrome, and myeloproliferative neoplasm (Hsieh et al. 2024; Rao et al. 2021), 1 study incorporated patients with acute myeloid leukemia (AML) (Chan et al. 2024), and 1 did not specify the HM type. Black participants made up between 2% and 22.2% of the total participants included in most of the studies. The 3 studies that looked at only multiple myeloma patients had the highest number of Black participants at 20.4% (Fossum et al. 2021), 20.44% (Al Hadidi et al. 2021) and 22.2% (Jackson et al. 2022). The study that had the lowest number of Black participants (2%) looked at patients with AML (Chan et al. 2024).

### Main findings of included publications

The 3 publications that focused on multiple myeloma patients reported findings of statistically significant differences in PC use between Black and White patients. The article by FossumFossum et al. (2021) which examined racial/ethnic disparities in the use of palliative radiotherapy showed that Black patients were 13% less likely to receive this treatment within 12 months of being diagnosed with multiple myeloma compared to non-Hispanic Whites (OR = 0.87, 95% CI = 0.83–0.90, *p* < .0001). In the article by (Al Hadidi et al. 2021), the researchers aimed to identify if the gaps in care for Hispanics and non-Hispanic Blacks with multiple myeloma had decreased over time. The study findings showed that non-Hispanic Blacks had lower odds of receiving PC consultations compared to Non-Hispanic Whites (OR = 0.91, 95% CI = 0.85–0.97, p = 0.01). Finally, the article by (Jackson et al. 2022) which evaluated the sociodemographic and hospital-level factors associated with PC utilization among hospitalized multiple myeloma patients reported that non-Hispanic Blacks were less likely to use PC compared to non-Hispanic Whites (adjusted OR = 0.86, 95% CI = 0.79–0.94).

One of the included publications reported similar findings in PC use between Black and White HM patients but did not add any statistical findings such as p-values or confidence intervals (Hsieh et al. 2024). For example, the researchers noted that the rate of PC use was higher in White patients compared to Black patients (56% vs 49%) and the use of PC was associated with lower hospital charges across all racial groups. In contrast, (Rao et al. 2021) reported something different in their study. Using a mixed-effects multivariable logistic regression model the authors reported that early billed PC services were rendered more frequently to Black patients when compared with White patients (OR = 1.45, 95% CI = 1.22–1.71, *p* < .001).

In 2 of the studies, race/ethnicity was not a significant predictor of the differences in PC use among patients with HM. The articles by (Chan et al. 2024) and (Han et al. 2019) acknowledged that, even though there were reported differences in PC use between Black and White HM patients, these differences could not be attributed to the participants’ race or ethnicity. Table 1 presents an overview of the included articles with their significant findings.

**Table 1.** Overview of included articles

Authors + year of publication	Study aim(s)	Study design	Study population	Characteristics of Blacks included in the study	HM type studied	Significant findings
Fossum et al. 2021	To explore whether there are racial/ethnic disparities in the use of palliative radiotherapy for Multiple Myeloma	Cohort study using data from National Cancer Database (NCDB) registry	Inpatients with MM receiving palliative radiotherapy	Non-Hispanic Blacks made up 30,027 (20.4%) of the total study participants.	Multiple myeloma (MM)	AAs were 13% less likely to receive palliative radiotherapy within 12 months of being diagnosed with MM when compared to NHWs (OR = 0.87, 95% CI = 0.83 − 0.90, p < .0001)
				−15,657 (52.05%) of the Non-Hispanic Blacks were females		
				−23,586 (78.55%) were between the ages of 55 and 75 years		
				−12,332 (41.24%) earned less than $38,000.		
Hsieh et al. 2024	To investigate SES factors associated with palliative care use among hospitalized patients with history of blood cancer at life’s end	Retrospective study using National Inpatient Sample (NIS) data from 2016 through 2019	Adult patients with at least one malignant hematologic diagnosis who died within a minimum of 3 days after hospitalization.	African Americans made up (1142 vs 7098 Caucasians	-Leukemia Lymphoma	Palliative care use was higher in Caucasians (56%) compared to African Americans (49%).
				African Americans represented 5710 (11%) of all hospitalizations.	Myeloma	Hospital charges were less for those receiving palliative care across all racial groups including African Americans
Han et al. 2019	To explore palliative care use in patients undergoing HSCT during hospitalization in the United States, including general prevalence, temporal trends, and predictors of PC use.	Retrospective study using the National Inpatient Sample (NIS) data from 2008 to 2014	Adult patients aged 18 years and older who underwent Hematopoietic Stem Cell Transplantation (HSCT) during hospitalization.	Not specified	Not specified	Race was not a significant predictor of palliative care use for patients undergoing HSCT.
						Among the 1,381 patients who had received palliative care, 9.12% were Black as compared with 77.80% White patients.
Chan et al. 2024	To assess if higher income and/or higher education increased the likelihood of utilization of palliative care in patients.	Retrospective cross-sectional study of de-identified hospital-based data from the National Cancer Database (NCDB) from 2004 to 2016.	Adult patients over the ages of 18 years, and had newly diagnosed with Acute Myeloid Leukemia	Blacks made up 2% (2824) of the total study population.	Acute Myeloid Leukemia	Black patients had a lower odd of receiving palliative care compared to White patients even though it wasn’t statistically significant (0.90, CI: 0.79 − 1.03, p-value- 0.13)
	To explore the impact of insurance status and Medicaid expansion on palliative care utilization.					
Al Hadidi et al. 2021	To identify if the gaps in care for Hispanics and NH-Blacks with multiple myeloma (MM) were decreasing over time.	Retrospective cross-sectional analysis of inpatient hospitalizations from 2008 to 2017 using the Nationwide Inpatient Sample (NIS)	Adults aged 18 years and over with an occurrence of MM in the discharge records.	Non-Hispanic Blacks accounted for 186, 846 (20.44%) of the total MM patients included in the study.	Multiple Myeloma	Non-Hispanic Blacks had lower odds of receiving palliative care consultations when compared with Non-Hispanic Whites (0.91, CI: 0.85 − 0.97, p-value − 0.01)
	To investigate characteristics of MM-related hospitalizations including utilization of MM therapies across different racial/ethnic groups.					
Rao et al. 2021	To describe the use of billed Palliative Care services among Medicare beneficiarieswith Hematological Malignancies in terms of frequency, trends over time, and characteristics of the associated encounters.	Cohort study using the National Cancer Institute’s (NCI) Surveillance, Epidemiology, and End Results (SEER)-Medicare linked database.	Medicare beneficiaries aged 66 years or older diagnosed with leukemia, lymphoma, myeloma, myelodysplastic syndrome (MDS), or myeloproliferative neoplasm (MPN) who died between 2001 and 2015	Blacks made up 6.7% (8146) of the total participants (120,741) included in the study.	Leukemia Lymphoma Myeloma Myelodysplastic syndrome (MDS) Myeloproliferative neoplasm (MPN)	Early billed palliative care services were offered more frequently to Blacks (1.45, CI: 1.22 − 1.71, p-value = < 0.001) compared with White individuals.
	To test the hypothesis that the receipt of early PC services (>30 days beforedeath) may be associated with less aggressive EOL care, increased hospice use, and decreased health care utilization as suggested by research in solid tumors.					
Jackson et al. 2022	To examine the sociodemographic and hospital-level factors associated with palliative care utilization among multiple myeloma (MM) patients using the largest in-hospital database in the US. -Explore prevalence trends in palliative care utilization among patients with MM between 2016 and 2018.	Cross-sectional study using National Inpatient Sample (NIS) data collected between 2016 and 2018	All hospitalizations with a diagnosis of multiple myeloma.	Non-Hispanic Blacks made up 22.2% (15,173) of the total participants included in the study.	Multiple myeloma	Non-Hispanic Blacks made up 21.3% (1127) of the total 5,289 patients who used palliative care. Non-Hispanic Blacks were less likely (adjusted odds ratio of 0.86; 95% CI: 0.79 − 0.94) to utilize palliative care when compared with Non-Hispanic Whites.

## Discussion

The purpose of this scoping review was to identify and describe what is known about PC use among Black HM patients in the US. This review has established that there is lower PC use among HM Black patients compared to their White counterparts (Al Hadidi et al. 2021; Fossum et al. 2021; Jackson et al. 2022). This ongoing inequity in the accessibility and utilization of PC among Black patients with HM makes it necessary for us to find creative ways to improve PC access and use within this community. One effective way would be to train current oncology nurses in providing some as aspects of PC, including symptom management, psychosocial support and advance care planning (Resick et al. 2020; Walling et al. 2017). This can be achieved through training programs that that promote integrating PC domains into everyday work routines (Artioli et al. 2019). Given their excellent clinical and collaborative abilities and cost-effective nature, nurses are well-positioned to bridge this gap (Walling et al. 2017). Studies have reported that nurses and nurse practitioners are effective in supporting the PC needs of oncology patients undergoing treatment (Lewis 2020; Mitchell et al. 2016). The addition of culturally trained PC nurse practitioners working as a part of a hematology-oncology team could influence the uptake of PC for the HM patient populations (Lewis 2020).

The representation of Black participants in the included studies, varied significantly depending on the type of HM being studied. We found that Black participants constituted between 2% and 22.2% of the study populations, with the highest representation observed in the studies focused solely on multiple myeloma patients (Al Hadidi et al. 2021; Fossum et al. 2021; Jackson et al. 2022). This was not surprising, given that multiple myeloma is known to disproportionately affect Black individuals more than other racial groups (Rajkumar et al. 2018). On the other hand, the lowest representation (2%) was in the study on AML patients (Chan et al. 2024), pointing to a potential underrepresentation of Black individuals in AML research. Studies report that misunderstandings about PC (Johnson et al. 2009), conflicting spiritual and cultural beliefs (Stockdill et al. 2023), and mistrust of the medical system due to historical unethical practices like the Tuskegee syphilis study (Hong et al. 2018) have all contributed to the reduced participation in research among Black individuals. Therefore, there is a pressing need to develop strategies to recruit and retain Black patients in PC research to promote equitable PC access and delivery.

Research on health care disparities exists in three phases: (1) establishing the presence of disparities; (2) understanding the causes and factors contributing to those disparities, and (3) developing and testing interventions to eliminate those disparities (Kilbourne et al. 2006). In our review, all the included publications focused on establishing evidence of disparities in PC use, as such there’s the need for more research in the other two phases. According to (Johnson 2013), research aimed at understanding the factors that contribute to the disparities in PC use should incorporate prospective, longitudinal studies that assess possible moderators and mediators of PC inequalities at several levels, including the patient, clinician, and health care system or organization. Additionally, these studies should also be done with an eye towards building testable interventions focused on improving PC delivery for this patient population. Instead of promoting a limited approach to PC delivery, it is important to design and evaluate care models that can consider the cultural beliefs, values, and preferences of this patient group (Johnson 2013). The creation and testing of these interventions should also be grounded in current and developing evidence in the field (Hanson et al. 2013).

## Strengths and limitations

This review highlights critical gaps that require further attention to improve the PC access and use among Black HM patients in the US. Our study’s strength lies in the comprehensive approach we used to identify and describe what is known about PC use among Black HM patients in the US. Despite our findings, some limitations should be noted. First, since this review focused on PC, we only included studies that were self-identified as PC, end of life care, or hospice care; however, we may have missed studies that used alternative phrases for PC, EOL, and hospice. In addition, bias could have crept into the review due to resolving the conflicts by discussion and not conducting dual independent data extraction.

## Conclusion

We conducted a scoping review to identify and describe what is known about PC use among Black HM patients. Most of the articles focused on documenting disparities in access to PC use rather than investigating the root causes of these disparities. Further research is needed to understand the determinants of these disparities and to also build testable interventions to improve PC use within this underserved population.

## Supporting information

Ansah et al. supplementary material 1 Ansah et al. supplementary material

Ansah et al. supplementary material 2 Ansah et al. supplementary material

## Supplementary material

The supplementary material for this article can be found at https://doi.org/10.1017/S1478951525000471.
