# Supplementary material for: Exploring the landscape of palliative care provision for black patients with hematologic cancers: A scoping review
Source: Palliat Support Care. 2025 Apr 22;23:e96. doi: 10.1017/S1478951525000471 (PMC13166668; doi:10.1017/S1478951525000471)
Supplement: Ansah et al. supplementary material 2 — Ansah et al. supplementary material [file S1478951525000471sup002.docx]

**Appendix. Search Strategies Owusu Ansah**

**Exploring the Landscape of Palliative Care Provision for Black Patients with Hematologic Cancers: A Scoping Review.**

**Librarian Searcher:** Leila Ledbetter

**Date:** October 24, 2024

**Database / Study Registry (including vendor/platform):** MEDLINE (PubMed) **Inclusive Date Coverage:** 1966 to present, and selected coverage of literature prior to that period

| **Set #** | **Search Strategy** | **Results** |
| --- | --- | --- |
| 1  *Black/ African-American* | ("Black or African American"[Mesh:NoExp] OR Minority Groups[Mesh:NoExp] OR Racial Groups[Mesh:NoExp] OR "African American"[tiab] OR "African Americans"[tiab] OR "African ancestry"[tiab] OR "Black American"[tiab] OR "Black Americans"[tiab] OR Blacks[TIAB] OR minorities[TIAB] OR minority[TIAB] OR "people of color"[tiab] OR "people of colour"[tiab] OR "person of color"[tiab] OR "person of colour"[tiab] OR race[TIAB] OR racial[TIAB] OR "afro-american"[tiab]) NOT ((africa[MESH] OR asia[MESH] OR australia[MESH] OR canada[MESH] OR central america[mesh] OR europe[MESH] OR Mexico[MESH] OR south america[MESH]) NOT (north america[MeSH:noexp] OR united states[MESH])) | 320,487 |
| 2  *Hematologic cancers* | "Hematologic Neoplasms"[Mesh] OR "Leukemia"[Mesh] OR "Lymphoma"[Mesh] OR "Multiple Myeloma"[Mesh] OR "blood cancer"[tiab] OR "blood cancers"[tiab] OR Leukemia[tiab] OR leukemias[tiab] OR leukaemia[tiab] OR leukaemias[tiab] OR lymphoma[tiab] OR lymphomas[tiab] OR "Multiple Myeloma"[tiab] OR "Multiple Myelomas"[tiab] OR "Hodgkin Disease"[tiab] OR "Non-Hodgkin"[tiab] OR "Non-Hodgkin's"[tiab] OR nonhodgkin[tiab] OR nonhodgkins[tiab] OR "myelodysplastic syndromes"[tiab] OR "Myelodysplastic syndrome"[tiab] OR "myeloproliferative diseases"[tiab] OR "myeloproliferative disease"[tiab] OR "Clonal eosinophilias"[tiab] OR "Clonal eosinophilia"[tiab] OR "Primary myelofibrosis"[tiab] OR "Transient myeloproliferative disease"[tiab] OR “mycosis fungoides”[tiab] OR “Sézary syndrome”[tiab] OR “Waldenström macroglobulinemia”[tiab] OR ((Hematologic[tiab] OR Hematological[tiab] OR haematologic[tiab] OR haematological[tiab] hematopoietic[tiab] OR myeloid[tiab] OR lymphoid[tiab]) AND (Cancer[tiab] OR cancers[tiab] OR cancerous[tiab] OR carcinoid[tiab] OR Tumor[tiab] OR tumors[tiab] OR tumorous[tiab] OR tumour[tiab] OR tumours[tiab] OR tumourous[tiab] OR Malignancy[tiab] OR malignancies[tiab] OR malignant[tiab] OR Neoplasm[tiab] OR neoplasms[tiab] OR neoplasia[tiab] OR neoplastic[tiab] OR Carcinoma[tiab] OR carcinomas[tiab] OR carcinomatous[tiab] OR sarcoma[tiab] OR sarcomas[tiab] OR Oncology[tiab] OR oncologic[tiab])) | 697,771 |
| 3  *Palliative care* | "palliative care"[mesh] OR "palliative medicine"[mesh] OR "Hospice and Palliative Care Nursing"[Mesh] OR "Hospice Care"[Mesh] OR "Terminal Care"[Mesh] OR palliative[tiab] OR palliation[tiab] OR "end of life"[tiab] OR "EOL care"[tiab] OR hospice[tiab] OR hospices[tiab] OR "Terminal care"[tiab] OR "Terminal medicine"[tiab] | 174,619 |
| 4 | #1 AND #2 AND #3 | 61 |

**Database / Study Registry (including vendor/platform):** Embase (Elsevier)

**Inclusive Date Coverage:** 1947 -

| **Set #** | **Search Strategy** | **Results** |
| --- | --- | --- |
| 1  *Black/ African-American* | ('african american'/exp OR 'african american' OR 'minority group'/exp OR 'minority group' OR 'people of color'/exp OR 'people of color' OR 'ancestry group'/exp OR 'ancestry group' OR 'black person'/exp OR 'black person' OR 'african american':ti,ab OR 'african americans':ti,ab OR 'african ancestry':ti,ab OR 'black american':ti,ab OR 'black americans':ti,ab OR blacks:ti,ab OR minorities:ti,ab OR minority:ti,ab OR 'people of color':ti,ab OR 'people of colour':ti,ab OR 'person of color':ti,ab OR 'person of colour':ti,ab OR race:ti,ab OR racial:ti,ab OR 'afro american':ti,ab) NOT (('africa'/exp OR africa OR 'asia'/exp OR asia OR 'australia and new zealand'/exp OR 'australia and new zealand' OR 'canada'/exp OR canada OR 'central america'/exp OR 'central america' OR 'europe'/exp OR europe OR 'mexico'/exp OR mexico OR 'south america'/exp OR 'south america') NOT ('north america'/exp OR 'north america' OR 'united states'/exp OR 'united states')) | 715,808 |
| 2  *Hematologic cancers* | 'hematologic malignancy'/exp OR 'leukemia'/exp OR 'lymphoma'/de OR 'multiple myeloma'/exp OR 'blood cancer':ti,ab OR 'blood cancers':ti,ab OR leukemia:ti,ab OR leukemias:ti,ab OR leukaemia:ti,ab OR leukaemias:ti,ab OR aleukemia:ti,ab OR aleukaemia:ti,ab OR lymphoma:ti,ab OR lymphomas:ti,ab OR hemoblastoma:ti,ab OR 'multiple myeloma':ti,ab OR 'multiple myelomas':ti,ab OR 'hodgkin disease':ti,ab OR 'non-hodgkin':ti,ab OR 'non-hodgkins':ti,ab OR nonhodgkin:ti,ab OR nonhodgkins:ti,ab OR 'myelodysplastic syndromes':ti,ab OR 'myelodysplastic syndrome':ti,ab OR 'myeloproliferative diseases':ti,ab OR 'myeloproliferative disease':ti,ab OR 'clonal eosinophilias':ti,ab OR 'clonal eosinophilia':ti,ab OR 'primary myelofibrosis':ti,ab OR 'transient myeloproliferative disease':ti,ab OR 'mycosis fungoides':ti,ab OR 'sezary syndrome':ti,ab OR 'waldenström macroglobulinemia':ti,ab OR ((hematologic:ti,ab OR hematological:ti,ab OR haematologic:ti,ab OR 'haematological hematopoietic':ti,ab OR myeloid:ti,ab OR lymphoid:ti,ab) AND (cancer:ti,ab OR cancers:ti,ab OR cancerous:ti,ab OR carcinoid:ti,ab OR tumor:ti,ab OR tumors:ti,ab OR tumorous:ti,ab OR tumour:ti,ab OR tumours:ti,ab OR tumourous:ti,ab OR malignancy:ti,ab OR malignancies:ti,ab OR malignant:ti,ab OR neoplasm:ti,ab OR neoplasms:ti,ab OR neoplasia:ti,ab OR neoplastic:ti,ab OR carcinoma:ti,ab OR carcinomas:ti,ab OR carcinomatous:ti,ab OR sarcoma:ti,ab OR sarcomas:ti,ab OR oncology:ti,ab OR oncologic:ti,ab)) | 1,141,859 |
| 3  *Palliative care* | 'palliative therapy'/exp OR 'Palliative Nursing'/exp OR 'Hospice Care'/exp OR 'Terminal Care'/exp OR palliative:ti,ab OR palliation:ti,ab OR 'end of life':ti,ab OR 'EOL care':ti,ab OR hospice:ti,ab OR hospices:ti,ab OR 'Terminal care':ti,ab OR 'Terminal medicine':ti,ab | 292,318 |
| 4 | #1 AND #2 AND #3 | 605 |
| 5 | #4 NOT ('editorial'/exp OR [editorial]/lim OR 'letter'/exp OR [letter]/lim OR 'note'/exp OR [note]/lim OR [conference abstract]/lim OR 'conference abstract'/exp OR 'conference abstract'/it) | 226 |

**Database / Study Registry (including vendor/platform):** Web of Science (Clarivate)

**Inclusive Date Coverage:** Core Collection: Science Citation Index Expanded--1900-present; Social Sciences Citation Index--1900-present; Arts & Humanities Citation Index--1975-present; Conference Proceedings Citation Index – Science--1990-present; Conference Proceedings Citation Index – Social Science & Humanities--1990-present; Book Citation Index – Science--2005-present; Book Citation Index – Social Sciences & Humanities--2005-present; Emerging Sources Citation Index--2017-present; Current Chemical Reactions--1985-present; Index Chemicus--1993-present

| **Set #** | **Search Strategy** | **Results** |
| --- | --- | --- |
| 1  *Black/ African-American* | ALL=(("Minority Groups" OR Racial Groups OR "African American" OR "African Americans" OR "African ancestry" OR "Black American" OR "Black Americans" OR Blacks OR minorities OR minority OR "people of color" OR "people of colour" OR "person of color" OR "person of colour" OR race OR racial OR "afro-american") ) | 1,235,479 |
| 2  *Hematologic cancers* | ALL=("blood cancer" OR "blood cancers" OR Leukemia OR leukemias OR leukaemia OR leukaemias OR lymphoma OR lymphomas OR "Multiple Myeloma" OR "Multiple Myelomas" OR "Hodgkin Disease" OR "Non-Hodgkin" OR "Non-Hodgkin's" OR nonhodgkin OR nonhodgkins OR "myelodysplastic syndromes" OR "Myelodysplastic syndrome" OR "myeloproliferative diseases" OR "myeloproliferative disease" OR "Clonal eosinophilias" OR "Clonal eosinophilia" OR "Primary myelofibrosis" OR "Transient myeloproliferative disease" OR “mycosis fungoides” OR “Sézary syndrome” OR “Waldenström macroglobulinemia” ) | 807,804 |
| 3  *Hematologic cancers* | ALL=( ((Hematologic OR Hematological OR haematologic OR haematological OR myeloid OR lymphoid) AND (Cancer OR cancers OR cancerous OR carcinoid OR Tumor OR tumors OR tumorous OR tumour OR tumours OR tumourous OR Malignancy OR malignancies OR malignant OR Neoplasm OR neoplasms OR neoplasia OR neoplastic OR Carcinoma OR carcinomas OR carcinomatous OR sarcoma OR sarcomas OR Oncology OR oncologic)) ) | 233,697 |
| 4 | #2 or #3 | 901,423 |
| 5  *Palliative care* | ALL=(palliative OR palliation OR "end of life" OR "EOL care" OR hospice OR hospices OR "Terminal care" OR "Terminal medicine") | 160,852 |
| 6 | #1 AND #4 AND #5 | 124 |

**Database / Study Registry (including vendor/platform):** CINAHL Complete (EBSCOhost)

**Inclusive Date Coverage:** 1937 -

| **Set #** | **Search Strategy** | **Results** |
| --- | --- | --- |
| S1  *Black/ African-American* | (MH "African Americans") OR (MH "Minority Groups") OR TI ("African American" OR "African Americans" OR "African ancestry" OR "Black American" OR "Black Americans" OR Blacks OR minorities OR minority OR "people of color" OR "people of colour" OR "person of color" OR "person of colour" OR race OR racial OR "afro-american") OR AB ("African American" OR "African Americans" OR "African ancestry" OR "Black American" OR "Black Americans" OR Blacks OR minorities OR minority OR "people of color" OR "people of colour" OR "person of color" OR "person of colour" OR race OR racial OR "afro-american") NOT((MH "Australia+") OR (MH "Canada+") OR (MH "Africa+") OR (MH "Asia+") OR (MH "Central America+") OR (MH "Europe+") OR (MH "Mexico") OR (MH "South America+") NOT (MH "North America+") OR (MH "United States+")) | 130,717 |
| S2  *Hematologic cancers* | (MH "Hematologic Neoplasms+") OR (MH "Leukemia+") OR (MH "Lymphoma+") OR (MH "Multiple Myeloma+") OR TI("blood cancer" OR "blood cancers" OR Leukemia OR leukemias OR leukaemia OR leukaemias OR lymphoma OR lymphomas OR "Multiple Myeloma" OR "Multiple Myelomas" OR "Hodgkin Disease" OR "Non-Hodgkin" OR "Non-Hodgkin's" OR nonhodgkin OR nonhodgkins OR "myelodysplastic syndromes" OR "Myelodysplastic syndrome" OR "myeloproliferative diseases" OR "myeloproliferative disease" OR "Clonal eosinophilias" OR "Clonal eosinophilia" OR "Primary myelofibrosis" OR "Transient myeloproliferative disease" OR “mycosis fungoides” OR “Sézary syndrome” OR “Waldenström macroglobulinemia”) OR AB("blood cancer" OR "blood cancers" OR Leukemia OR leukemias OR leukaemia OR leukaemias OR lymphoma OR lymphomas OR "Multiple Myeloma" OR "Multiple Myelomas" OR "Hodgkin Disease" OR "Non-Hodgkin" OR "Non-Hodgkin's" OR nonhodgkin OR nonhodgkins OR "myelodysplastic syndromes" OR "Myelodysplastic syndrome" OR "myeloproliferative diseases" OR "myeloproliferative disease" OR "Clonal eosinophilias" OR "Clonal eosinophilia" OR "Primary myelofibrosis" OR "Transient myeloproliferative disease" OR “mycosis fungoides” OR “Sézary syndrome” OR “Waldenström macroglobulinemia”) | 87,757 |
| S3  *Hematologic cancers* | TI((Hematologic OR Hematological OR haematologic OR haematological hematopoietic OR myeloid OR lymphoid) AND (Cancer OR cancers OR cancerous OR carcinoid OR Tumor OR tumors OR tumorous OR tumour OR tumours OR tumourous OR Malignancy OR malignancies OR malignant OR Neoplasm OR neoplasms OR neoplasia OR neoplastic OR Carcinoma OR carcinomas OR carcinomatous OR sarcoma OR sarcomas OR Oncology OR oncologic)) OR AB((Hematologic OR Hematological OR haematologic OR haematological hematopoietic OR myeloid OR lymphoid) AND (Cancer OR cancers OR cancerous OR carcinoid OR Tumor OR tumors OR tumorous OR tumour OR tumours OR tumourous OR Malignancy OR malignancies OR malignant OR Neoplasm OR neoplasms OR neoplasia OR neoplastic OR Carcinoma OR carcinomas OR carcinomatous OR sarcoma OR sarcomas OR Oncology OR oncologic)) | 18,146 |
| S4 | S2 or S3 | 96,224 |
| S5  *Palliative care* | (MH "Palliative Medicine") OR (MH "Palliative Care") OR (MH "Palliative Care Nursing") OR (MH "Hospice Care") OR (MH "Hospice Nursing") OR (MH "Hospice Patients") OR (MH "Terminal Care+") OR TI(palliative OR palliation OR "end of life" OR "EOL care" OR hospice OR hospices OR "Terminal care" OR "Terminal medicine") OR AB(palliative OR palliation OR "end of life" OR "EOL care" OR hospice OR hospices OR "Terminal care" OR "Terminal medicine") | 101,770 |
| 4 | S1 AND S4 AND S5 | 32 |

Total number of citations before de-duplication: 443
